# From impact to recovery: tracking mild traumatic brain injury with MRI—a pilot study and case series

**DOI:** 10.1136/bmjsem-2024-002010

**Published:** 2024-08-01

**Authors:** Xuan Vinh To, Paul Cumming, Fatima Nasrallah

**Affiliations:** 1 The Queensland Brain Institute, The University of Queensland, St. Lucia, Queensland, Australia; 2 Department of Nuclear Medicine, Inselspital University Hospital Bern, Bern, Switzerland; 3 School of Psychology and Counselling, Queensland University of Technology, Brisbane, Queensland, Australia; 4 The Centre for Advanced Imaging, The University of Queensland, Brisbane, Queensland, Australia

**Keywords:** Concussion, Trauma, Rehabilitation, Brain

## Abstract

**Background:**

Diagnosis and recovery tracking of mild traumatic brain injury (mTBI) is often challenging due to the lack of clear findings on routine imaging techniques. This also complicates defining safe points for returning to activities.

**Hypothesis/purpose:**

Quantitative susceptibility mapping (QSM) can provide information about cerebral venous oxygen saturation (CSvO_2_) in the context of brain injury. We tested the prediction that these imaging modalities would enable the detection of changes and recovery patterns in the brains of patients with mTBI.

**Study design:**

In a case-control study, we recruited a cohort of 24 contact sport athletes for baseline QSM and resting-state functional MRI (rs-fMRI) scanning. Two of those who subsequently experienced head impact with significant post-injury symptoms underwent scans at 3, 7, 14 and 28 days post-injury; one had a boxing match without classical mTBI symptoms were also followed-up on.

**Results:**

The cohort baseline QSM measurements of the straight sinus were established. The two injured athletes with post-impact symptoms consistent with mTBI had susceptibility results at days 3 and 7 post-impact that fell below the 25th percentile of the baseline values. The per cent amplitude fluctuation quantified from rs-fMRI agreed with the susceptibility trends in the straight sinus.

**Conclusion:**

QSM holds promise as a diagnostic tool for tracking mTBI progression or recovery in contact sport head injury.

**WHAT IS ALREADY KNOWN ON THIS TOPIC**

- Quantitative susceptibility mapping (QSM) can provide a method to quantify the cerebral venous oxygen saturation (CSvO_2_) in the major veins. Higher CSvO_2_ can be an indicator of brain injury and thus a potential objective measurement of recovery after a mild traumatic brain injury (mTBI).

**WHAT THIS STUDY ADDS**

- The results showed a significant decrease in QSM signal indicative of increased CSvO_2_ and brain injury in two athletes with symptoms consistent with mTBI between days 3 and 7 post-mTBI, but not in an athlete with some head impacts but no symptoms. This change normalised at around day 14. A normative dataset of 24 contact sport athletes showed that the susceptibility values of the mTBI participants during the day 3–7 period were below the 25th percentile of the normative baseline dataset.

**HOW THIS STUDY MIGHT AFFECT RESEARCH, PRACTICE OR POLICY**

- The study suggests the value of QSM measurement of CSvO_2_ in the straight sinus for tracking mTBI recovery temporally. Further studies into the evolution of the CSvO_2_ values temporally in mTBI cases and the test-retest reliability of baselines are required.

## Introduction

 Mild traumatic brain injury (mTBI) is a pathophysiological process manifesting as a rapid-onset transient neurological disturbance as a consequence of head physical trauma, excluding more severe forms of TBI (ie, those with obvious lesions in or penetrations into the brain).^1^ mTBI is a prevalent concern in contact sports and represents a significant public health challenge: 100–300 hospital treatments per 100 000 people are attributed to mTBI annually, as highlighted in studies across various regions and countries.^2^ Notably, athletes, military personnel and domestic violence victims have a disproportionate risk of experiencing repeated mTBIs. The cumulative effect of multiple mTBIs within a short period can lead to exacerbated brain damage.^3^,^6^ This results in the consensus that patients with mTBI should only return to high-risk activities upon complete recovery; though, objective criteria for complete recovery remain challenging.^7^ Currently, clinicians predominantly rely on subjective measures like symptom tracking and neuropsychological tests, which may not fully capture the nuances of mTBI.^7^ Thus, in the context of sports medicine, there are no objective grounds for determining how long asymptomatic patients with mTBI should withdraw from risky activities.

An immediate concern for the management of patients with mTBI in the emergency department lies in the detection of complications such as skull fractures or intracranial haemorrhages and in the management of delayed intracranial haemorrhages not detected with initial structural imaging.^8^ Thus, routine clinical imaging for patients with mTBI serves mainly for the exclusion of any severe neuropathology that would call for reclassification of the injury. Neuroimaging, particularly MRI, has emerged as a potential avenue for more precise mTBI diagnostics,^9^ but they are not standard for mTBI assessment. mTBI literature of these methods typically showed population-based statistical analyses, which require extensive normative data (ie, data from a large and diverse number of healthy controls) for accurate interpretation.^9^ For instance, NeuroQuant, the only US Food and Drug Administration-approved MRI-based diagnostic method for mTBI, uses a database of over 100 000 healthy controls’ structural MRI scans.^10^ While this database affords an excellent representation of normative population results, it is notable that even the simplest MRI method, which is structural MRI, can be affected by variations in scanner software and hardware, which may shift the quantified metrics by as much as 5%^11 12^; most purported effects detectable on MRIs have a much smaller effect size.

Amidst these challenges, quantitative susceptibility mapping (QSM), a more advanced derivative of susceptibility weighted imaging (SWI), has emerged as a promising MRI modality for mTBI monitoring. QSM records perturbations in the phase information of a multiecho scan to infer and model tissue magnetic properties at the voxel level.^13^ Cerebral venous oxygen saturation (CSvO_2_) can be measured using QSM using a few modelling assumptions.^14^ An elevated CSvO_2_ score, indicating a ‘luxury perfusion’ state or decreased oxygen consumption in the brain, is associated with brain injury.^15^ The relationship between CSvO_2_ and a given vein’s susceptibility is expressed as^14^:

$$\Delta \chi =\Delta \chi_{do}\times HCT\times \left(1-{CSvO}_{2}\right)$$

where Δχ is the susceptibility difference between the venous blood and the surrounding tissue; Δχdo is the susceptibility difference per unit of haematocrit between fully deoxygenated and fully oxygenated blood, and HCT is the individual’s haematocrit fraction. QSM susceptibility maps are reconstructed with an inherent offset of unknown scale, which precludes direct intersubject comparison without a zero reference; the optimal zero referencing strategy for QSM remains an active area of research.^16^ The approach to quantifying CSvO_2_ from QSM circumvents the zero-referencing issue in other QSM applications.

Resting-state functional MRI (rs-fMRI) measures the synchronous fluctuation of blood oxygenation level-dependent (BOLD) signals across different brain regions in awake subjects undergoing a rs-fMRI scan.^17^ QSM CSvO_2_ measurements initially served to validate BOLD-dependent rs-fMRI^14^ their concurrent measurement may be mutually complementary. In this preliminary case series, we examined the capability of QSM and rs-fMRI to detect and track mTBI recovery longitudinally with QSM and rs-fMRI.

## Materials and methods

The institutional Human Research Ethics Committees of the University of Queensland approved this study (approval number 2021/HE002696). 24 contact sport athletes (aged 26±4 years) were recruited and gave informed consent for their participation in the study and release of de-identified information for publication purposes. Each underwent a pre-injury baseline scanning session. Three participants received a sports-related head impact during the follow-up study period (approximately 4 years) and were rescanned between 3 and 42 days post-impact, most commonly at 3, 7, 14 and 28 days post-impact. T1-weighted structural imaging, rs-fMRI and three-dimensional multiecho gradient echo MRI (mGRE-MRI) were acquired. All images were registered together across participants and modalities. mGRE-MRI was fitted for QSM and rs-fMRI data were artefact-cleaned with group-information-guided independent component analysis^18^ and analysed with per cent amplitude fluctuation (PerAF).^19^

Since the study participants were otherwise fit and healthy (except for contact sport exposure and possible earlier history of head impact/injury), we assumed that their HCTs did not differ significantly from one another and had not changed significantly post-impact. We hypothesised that Δχ and CSvO_2_ would follow an inversely proportional relationship.

## Results

**Figure 1:**
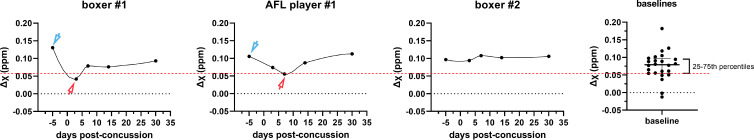
Susceptibility difference (Δχ) between the straight sinus and background brain tissue measured in the three athletes who returned for follow-up scans after head impact (symptomatic boxer #1, symptomatic AFL player #1 and asymptomatic boxer #2) and the mean baseline scans of all 24 participants. The day 3 and day 7 post-impact Δχ of boxer #1 and AFL player #1 were below the 25th percentile of the baseline values (red arrows below red dashed line).

**Figure 2:**
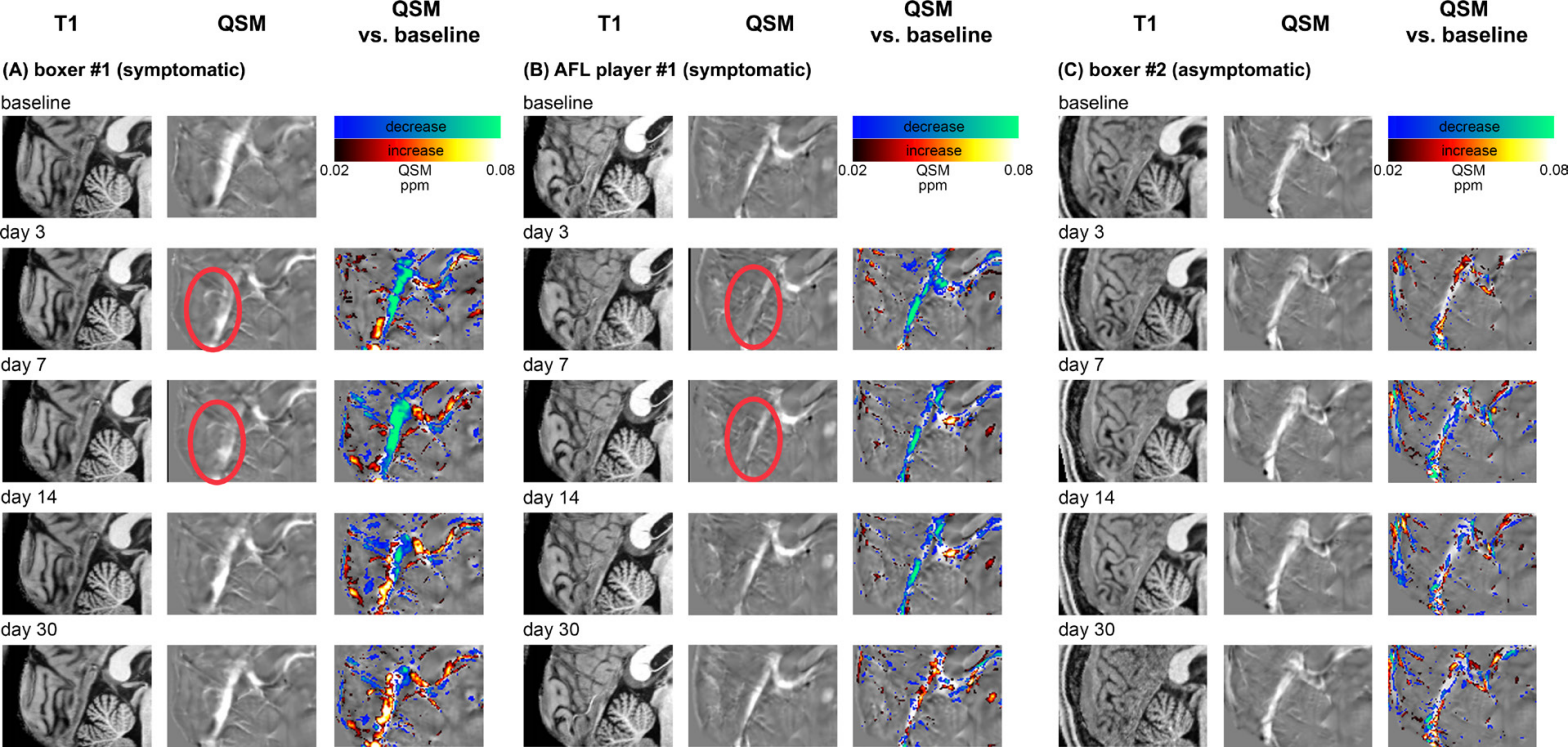
The evolution of the T1-weighted structural images, QSM and the change in the susceptibility maps in the three participants who returned for follow-up scans, all relative to individual baseline values. QSM, quantitative susceptibility maps.

The Δχ measured between the straight sinus ROI and the background ROI from the baseline scans of all 24 participants gave a range of values (figure 1A – baselines). The two participants (boxer #1 and AFL player #1) returned with significant symptoms after a head impact (table 1). The susceptibility difference (Δχ) at days 3–7 post-impact fell below the 25th percentile of the mean baseline Δχ values (figure 1A, red arrows). The initially lower Δχ values had apparently normalised by day 14 post-impact. The decreased susceptibility at days 3 and 7 post-impact was not statistically significant (paired t-test vs own baseline: p=0.25. t-test vs all baselines: p=0.80). Among the symptomatic returnees, boxer #1 had a longer symptomatic duration than AFL player #1. The Δχ of boxer #1 did not return to his pre-injury baseline (figure 1A, blue arrows), while AFL player #1 apparently had normalised Δχ by day 30 post-injury. Boxer #2 returned for follow-up scans after a competition fight; his complaint of facial skin hypersensitivity was not a post-concussion/mTBI symptom, and he did not manifest any decrease in Δχ post-impact. To visual inspection, the registered T1 structural image of the three follow-up participants did not show any particularly remarkable features at any time point (figure 2—T1 images). On the other hand, the QSM images of boxer #1 and AFL player #1 showed a noticeable decrease in the contrast of the straight sinus relative to the tissue background at days 3 and 7 scans (figure 2A,B—days 3 and 7).

**Table 1:** Details of the participants returning for follow-up assessments following head impacts

Participants' number	Sport	Age at impact (years)	Recalled prior concussion/mTBI	Baseline to impact (months)	Mechanism of head impact	Days post-impact (days)	Clinical diagnosis/self-reported symptoms
17	Boxing	26	1×1 year prior to study’s baseline	2	Boxing sparring and punch to the head	On the day	Dizziness, ‘dazzed’, ‘room spinning’ feeling
						3	Dizziness, loss of balance, brain fog, difficulty concentrating at work, nausea and headaches
						7	Dizziness, loss of balance, brain fog, difficulty concentrating at work, nausea and headaches
						14	Dizziness, loss of balance, brain fog, difficulty concentrating at work, nausea and headaches
						30	Recovered
25	Australian football league	31	1×14 years and 3×8 years prior to study’s baseline	3	Football and head impact	On the day	10–20 min loss of consciousness
						3	Headache, nausea, difficult of thinking, and sleepiness
						7	Severe headaches at days 9–10 post-impact, participant consulted with the general practitioner and told to rest
						14	None
						30	None
29	Boxing	20	None	0.75	Boxing match, multiple hits to head and face	On the day	Facial skin hypersensitivity
						3	Facial skin hypersensitivity
						7	None
						14	None
						30	None

All of 24 participants were males.

mTBImild traumatic brain injury

**Figure 3:**
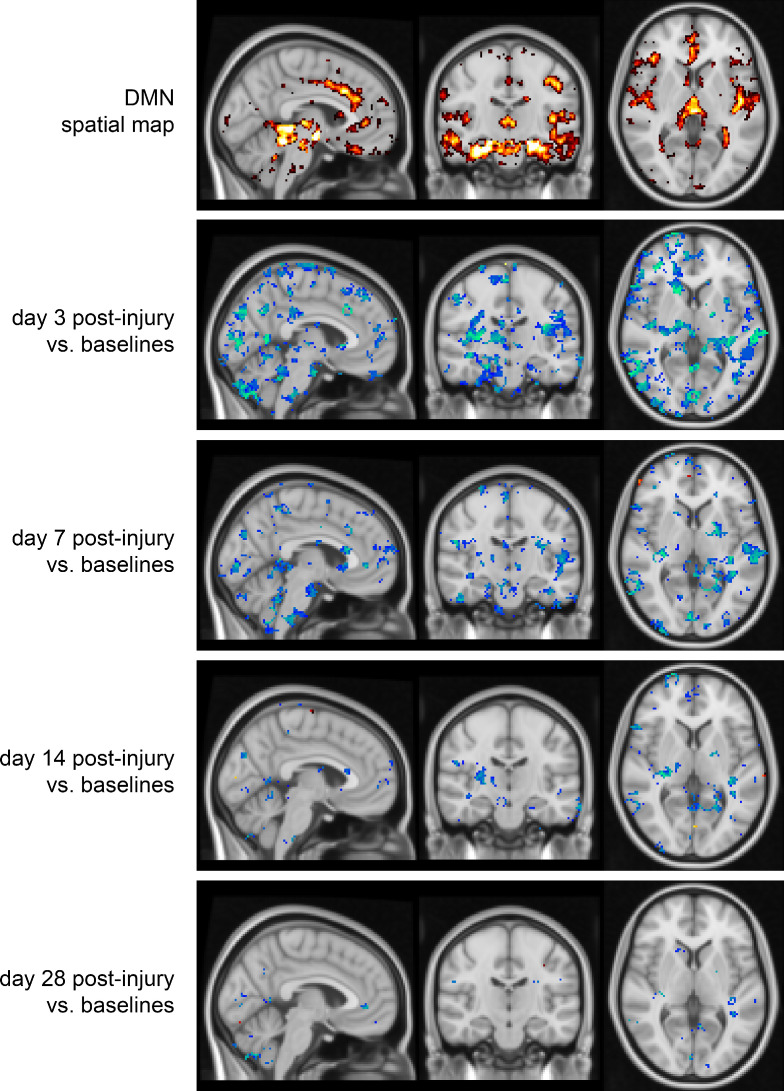
Voxel-wise statistical maps comparing the PerAF at different time points post-injury in the three follow-up cases to the mean baseline PerAF map of all 24 participants. First row includes all scans averaged spatial map of the independent component of the group-information-guided independent component analysis represents the DMN. DMN, Default Mode Network; PerAF, percent amplitude fluctuation;

PerAF analysis in the two mTBI cases also showed significant decreases at days 3 and 7 post-impact compared with the baselines (figure 3).

## Discussion

The straight sinus starts at the confluence of the inferior sagittal sinus and the great vein of Galen, thereby serving as the main venous drainage for the forebrain. In principle, QSM changes in the straight sinus will consequently reflect whole-hemispheric changes in cerebral oxygen metabolism. In previous QSM studies, groups of patients with mTBI imaged prior to day 15 post-injury had significantly lower straight sinus susceptibility compared with controls.^20 21^ Similar imaging performed on sports-related concussed amateur Australian football players at day 13 post-injury showed a trend towards a significant reduction in the female (six concussed vs seven controls), but not in the male participants (seven concussed vs nine controls).^22^ These findings and the relative simplicity of the quantification of QSM suggest that QSM can be clinically feasible for tracking mTBI recovery. Rs-fMRI provided cross-modality consistency.

While this is the first study with longitudinal data, there were only three participants with a full-time course, of whom only two had likely mTBI. Power analysis, using the mean (±SD) (0.047±0.043 ppm) of the susceptibility decrease in the straight sinus of the two concussion cases, predicts that 11 cases with pre-mTBI and post-mTBI scans would suffice for detecting significance using a paired t-test with a 0.05 Type I error probability and 0.95 power. Test-retest reliability among healthy controls and intervendor and intersite variations will need to be assessed. While individualised baseline recordings may not be generally practical for most people, we note that populations with a significant repeated mTBI risk (ie, contact sport athletes or military personnel) might have sufficiently predictable occupation cycles to justify the routine recording of individualised baselines, for example, pre-season and pre-deployment scans. This approach may enable individualised baselines and post-mTBI scans done with identical scanners and sequences.

In conclusion, we present promising results. The longitudinal QSM of the straight sinus tracked the time course of recovery of cerebral oxygen metabolism post-mTBI relative to individual and cohort baseline measurements. QSM indicated elevated blood oxygenation in the straight sinus post-mTBI, which can be a result of increased cerebral blood perfusion or decreased cerebral blood oxygen consumption. This application of QSM can be used as either a comparison against a set of normative controls or as individualised baselines.

## supplementary material

10.1136/bmjsem-2024-002010 online supplemental file 1
