# Supplementary material for: From impact to recovery: tracking mild traumatic brain injury with MRI—a pilot study and case series
Source: BMJ Open Sport Exerc Med. 2024 Aug 1;10(3):e002010. doi: 10.1136/bmjsem-2024-002010 (PMC11298751; doi:10.1136/bmjsem-2024-002010)
Supplement: online supplemental file 1 [file bmjsem-10-3-s001.pdf]

Supporting Information for

From impact to recovery: tracking mild traumatic brain injury with magnetic resonance imaging.

Xuan Vinh To[1], Paul Cumming[3,4], and Fatima Nasrallah[1,2*]

[1] The Queensland Brain Institute, The University of Queensland, St Lucia, Australia
[2] The Centre for Advanced Imaging, The University of Queensland, St Lucia, Australia
[3] Department of Nuclear Medicine, Bern University Hospital, Bern, Switzerland
[4] School of Psychology and Counselling, Queensland University of Technology, Brisbane, Australia
* Author for correspondence:

Dr. Fatima Nasrallah
Mailing address: The Queensland Brain Institute, The University of Queensland
Building 79, Upland Road, Saint Lucia, Brisbane, Queensland, Australia 4072
Phone: +61 7 33460322
Email: f.nasrallah@uq.edu.au

This PDF file includes:

Supporting text
SI References

Supporting Information Text

Participants.

The institutional Human Research Ethics Committees (approval number 2021/HE002696) approved this study. All participants gave written informed consent. The participants were recruited through advertisements and flyers at the local sporting clubs. We recruited 24 contact sport athletes, each of whom underwent a pre-injury baseline scanning session. Inclusion criteria for the participants included: 18–40 years of age, 20/20 vision at baseline assessment, be of general good health at the time of baseline assessment, and no prior history of moderate-severe Traumatic Brain Injury (TBI), epilepsy, recent history of alcohol or drug abuse, or other medical conditions, cancer, mental, neurological illness, or prescription medication.

Three participants received a sports-related head impact during the follow-up study period and were re-scanned between 3- and 42- days post-impact, most commonly at 3-, 7-, 14- and 28-day post-impact. “Sports-related head impact” here is defined as any impact to the head occurring during the participants’ sports-related activity, including training or matches, regardless of whether the impact results in concussion diagnosis or symptoms. The MR scanning protocol included T1-weighted structural imaging, Quantitative Susceptibility Mapping (QSM), and rs-fMRI sequences.

The chosen follow-up timepoints were chosen according to the consensus on sports-related concussion at the time of the study conception, e.g. “For most injured athletes, cognitive deficits, balance and symptoms improve rapidly during the first 2 weeks after injury. Many past studies, particularly those published before 2005, concluded that most athletes recover from SRC and return to sport within 10 days” [1]. The timepoints were chosen out of logistical considerations for the participants’ schedule and for likelihoods to capture the post-injury dynamics of the pre-injury period to the symptomatic and symptoms recovery post-injury periods.

MRI scans

Participants were scanned on a 7T Siemens Magnetom system with Syngo MR E12 software and 32-channel head coil. The T1-weighted (T1w) structural scan was acquired with a Magnetization Prepared - RApid Gradient Echo (MP2RAGE) sequence with the following parameters: TR/TE = 4300/2.45 ms, matrix size = 256 × 300 × 320, and effective voxel resolution = 0.75 mm isometric). The QSM data were acquired using a 3D multi-echo gradient echo MRI (mGRE-MRI) sequence with the following parameters: Repetition Time (TR) = 36 ms, matrix size = 238 × 272 × 160, effective resolution = 0.75 mm isometric, 9 echoes with min Echo Time (TE)/ΔTE/maxTE = 3.15/3.15/28.35 ms, flip angle = 15°. Resting-state functional Magnetic Resonance Imaging (rs-fMRI) was acquired using an echo planar free induction decay (EP-FID) sequence with the following parameters TR/TE = 586/23 ms, matrix size = 104 × 104 × 55, effective resolution = 2 × 2 × 2.2 mm, and 580 temporal volumes.

Image processing

QSM reconstruction: Skull-stripping was performed using FSL BET [2] on each of the QSM magnitude images at each echo; the brain mask based on the 2nd echo (TE = 6.3 ms) proved to be the most satisfactory in terms of accurately delineate brain and non-brain tissue through visual inspection. Therefore, after rescaling to the range -π – π, the phase image was masked using the 2nd echo brain mask. Resultant masked phase data from mGRE-MRI scans were used to construct QSM images with the STI Suite (v.3.0) [3]. Raw phase data were unwrapped via Laplacian-based phase unwrapping with a padding size of 12 [4]. Background phase removal was performed using the V-SHARP method [5] with a filter size of 6. The inverse QSM problem was solved from the local tissue phase values using the iLSQR method.[3,6] This QSM pipeline had proved robust in a previous application [7]. We averaged the constructed QSM from all echoes to create a mean QSM map.

Rs-fMRI: Rs-fMRI data were corrected for slice-timing using FSL’s slicetimer, and motion-corrected using FSL’s mcflirt. The resultant slice-timing and motion-corrected rs-fMRI data were averaged to create a spatial representation of the MRI data in the EP-FDI space. This spatial representation image was N4ITK bias-field corrected [8] through the implementation in Advanced

Normalization Tool (ANTs v.2.3.4) [9]. The motion-corrected rs-fMRI data were bandpass-filtered at 0.008 – 0.85 Hz and Gaussian filtered with a full-width at half-maximum kernel of 4 mm.

Image registration: T1w structural images and the magnitude image of the mGRE-MRI at TE = 6.3 ms were corrected for bias field, and then segmented into probabilistic white matter (WM), grey matter (GM), cerebro-spinal fluid (CSF), skull, and "other tissue" maps using SPM12 (https://www.fil.ion.ucl.ac.uk/spm/software/spm12/). We summated and binarized the WM, GM, and CSF probabilistic maps to generate a brain mask in the T1w structural image space. The processed and skull-stripped magnitude images of the mGRE-MRI at TE = 6.3 ms were rigidly registered to the skull-stripped T1w structural image.

We accomplished spatial normalisation or image registration of the T1w structural images to a common space using an iterative image registration and study-specific template creation process as implemented in ANTS's antsMultivarateTemplateConstruction2.sh, with the MNI152-0.5 mm template serving as the initial target. We registered the generated template to the MNI152-0.5 mm template, thus ensuring that any image registered to the study-specific template would be in the MNI152 space. Thus, we warped individual QSM maps to the common space by concatenating the mGRE-MRI to T1w registration with the individual's T1w structural image registration to the common space.

The processed spatial representation images in EP-FID space were also normalised to a common space through an iterative image registration and study-specific EP-FID template creation process. Registration from EP-based images to EP-based templates can serve for faster registration without loss in accuracy [10]. The study-specific EP-FID template was also registered to the MNI152 space.

Correlates of cerebral venous oxygen saturation ($CSvO_2$) from QSM: We subtracted registered post-impact QSM maps by individual baseline QSM map to give a Δ-QSM map for the three participants who we re-scanned after a head impact. After averaging the registered QSM maps from all participants at baseline, we used a region competition snakes segmentation [11] available in ITK-SNAP (v.3.8) to construct a region-of-interest (ROI) for the straight sinus. We also defined a 20 mm diameter spherical ROI for quantification of the background brain tissue susceptibility.

The relationship between $CSvO_2$ and a given vein's susceptibility is expressed as [12]:

$$\Delta\chi = \Delta\chi_{do} \times HCT \times (1 - CSvO_2)$$

where $\Delta\chi$ is the susceptibility difference between the venous blood and the surrounding tissue, which was quantified using the straight sinus ROIs defined as above. $\Delta\chi_{do}$ is the susceptibility difference per unit of haematocrit between fully deoxygenated and fully oxygenated blood, and HCT is the individual's haematocrit fraction. We performed the scans on otherwise (except for contact sport exposure and potential history of head impact/injury) fit and healthy athletes. Therefore, we assumed that their HCTs did not differ significantly from one another and had not changed after head impact. As such, we treated the $\Delta\chi_{do} \times HCT$ term as a constant across participants. Hence, we hypothesized that $\Delta\chi$ and $CSvO_2$ followed an inversely proportional relationship, such that a smaller $\Delta\chi$ would reflect higher $CSvO_2$, i.e., higher venous blood oxygenation, which in turn indicates lower oxygen consumption or "luxury perfusion" in the injured upstream brain. We quantified $\Delta\chi$ values for each participant's post-impact time point and across baseline scans for all participants.

Rs-fMRI analysis: The registered rs-fMRI were decomposed into independent components using Group-Information-Guided Independent Component Analysis (GIG-ICA) [13]. The number of independent components was set to 74, and GIG-ICA was used to perform artefact removal from the rs-fMRI data [14]. Percent amplitude fluctuation (PerAF) [15] was used to quantify the spontaneous brain activity from the artefact-cleaned rs-fMRI data via the RESTplus toolbox (http://www.restfmri.net) [16]. PerAF has been shown to be a valid and more reliable and straightforward measure [15] of spontaneous brain activity than the more traditional approach of amplitude of low-frequency fluctuation (ALFF) [17,18]. We made a voxel-wise comparison of post-head impact PerAF maps of the three participants who had an impact during the follow-up period

with the mean baseline PerAF map for all 25 participants. The statistical results were corrected for multiple correction using threshold-free cluster enhancement (TFCE) [19] at P value < 0.05.

SI References

1 McCrory P, Meeuwisse W, Dvořák J, *et al.* Consensus statement on concussion in sport—the 5th international conference on concussion in sport held in Berlin, October 2016. *Br J Sports Med*. 2017;51:838–47.

2 Smith SM. Fast robust automated brain extraction. *Hum Brain Mapp*. 2002;17:143–55.

3 Li W, Wang N, Yu F, *et al.* A method for estimating and removing streaking artifacts in quantitative susceptibility mapping. *Neuroimage*. 2015;108:111–22.

4 Li W, Wu B, Liu C. Quantitative susceptibility mapping of human brain reflects spatial variation in tissue composition. *Neuroimage*. 2011;55:1645–56.

5 Schweser F, Deistung A, Lehr BW, *et al.* Quantitative imaging of intrinsic magnetic tissue properties using MRI signal phase: An approach to in vivo brain iron metabolism? *Neuroimage*. 2011;54:2789–807.

6 Wang N, Cofer G, Anderson RJ, *et al.* Accelerating quantitative susceptibility imaging acquisition using compressed sensing. *Phys Med Biol*. 2018;63. doi: 10.1088/1361-6560/aaf15d

7 Schweser F, Robinson SD, de Rochefort L, *et al.* An illustrated comparison of processing methods for phase MRI and QSM: removal of background field contributions from sources outside the region of interest. *NMR Biomed*. 2017;30. doi: 10.1002/nbm.3604

8 Tustison NJ, Avants BB, Cook PA, *et al.* N4ITK: Improved N3 Bias Correction. *IEEE Trans Med Imaging*. 2010;29:1310–20.

9 Avants BB, Tustison NJ, Stauffer M, *et al.* The Insight ToolKit image registration framework. *Front Neuroinform*. 2014;8:1–13.

10 Dohmatob E, Varoquaux G, Thirion B. Inter-subject registration of functional images: Do we need anatomical images? *Front Neurosci*. 2018;12:1–11.

11 Ho S, Cody H, Gerig G. SNAP : A Software Package for User-Guided Geodesic Snake Segmentation. *6th International Conference on Medical Image Computing and Computer Assisted Intervention*. Montreal, Canada 2003. https://www.sci.utah.edu/~gerig/publications/MICCAI03-Ho-snap.pdf

12 Haacke EM, Lai S, Reichenbach JR, *et al.* In vivo measurement of blood oxygen saturation using magnetic resonance imaging: A direct validation of the blood oxygen level-dependent concept in functional brain imaging. *Hum Brain Mapp*. 1997;5:341–6.

13 Du Y, Fan Y. Group information guided ICA for fMRI data analysis. *Neuroimage*. 2013;69:157–97.

14 Du Y, Allen EA, He H, *et al.* Artifact removal in the context of group ICA: A comparison of single-subject and group approaches. *Hum Brain Mapp*. 2016;37:1005–25.

15 Jia XZ, Sun JW, Ji GJ, *et al.* Percent amplitude of fluctuation: A simple measure for resting-state fMRI signal at single voxel level. *PLoS One*. 2020;15:1–15.

16 Jia XZ, Wang J, Sun HY, *et al.* RESTplus: an improved toolkit for resting-state functional magnetic resonance imaging data processing. *Sci Bull*. 2019;64:953–4.

17 Zang YF, Yong H, Chao-Zhe Z, *et al.* Altered baseline brain activity in children with ADHD revealed by resting-state functional MRI. *Brain Dev*. 2007;29:83–91.

18 Zou QH, Zhu CZ, Yang Y, *et al.* An improved approach to detection of amplitude of low-frequency fluctuation (ALFF) for resting-state fMRI: Fractional ALFF. *J Neurosci Methods*. 2008;172:137–41.

19 Smith SM, Nichols TE. Threshold-free cluster enhancement: Addressing problems of smoothing, threshold dependence and localisation in cluster inference. *Neuroimage*. 2009;44:83–98.
